# Dehydrothyrsiferol Against Cutaneous Leishmaniasis: Treatment Outcome in a Murine Model

**DOI:** 10.3390/md23010013

**Published:** 2024-12-28

**Authors:** Atteneri López-Arencibia, Carlos J. Bethencourt-Estrella, Desirée San Nicolás-Hernández, Rubén L. Rodríguez-Expósito, Angélica Domínguez-de-Barros, Lizbeth Salazar-Villatoro, Maritza Omaña-Molina, Francisco Cen-Pacheco, Ana R. Díaz-Marrero, José J. Fernández, Elizabeth Córdoba-Lanús, Jacob Lorenzo-Morales, José E. Piñero

**Affiliations:** 1 Instituto Universitario de Enfermedades Tropicales y Salud Pública de Canarias (IUETSPC), Universidad de La Laguna (ULL), Avenida Astrofísico Francisco Sánchez s/n, 38206 La Laguna, Spain; cbethene@ull.edu.es (C.J.B.-E.); dsannico@ull.edu.es (D.S.N.-H.); rrodrige@ull.edu.es (R.L.R.-E.); angelica4arealejos@gmail.com (A.D.-d.-B.); acordoba@ull.edu.es (E.C.-L.);; 2 Consorcio Centro de Investigación Biomédica en Red M.P. de Enfermedades Infecciosas (CIBERINFEC), Instituto de Salud Carlos III, 28006 Madrid, Spain; 3 Departamento de Obstetricia y Ginecología, Pediatría, Medicina Preventiva y Salud Pública, Toxicología, Medicina Legal y Forense y Parasitología, Universidad de La Laguna (ULL), 38200 La Laguna, Spain; 4 Departamento de Infectómica y Patogénesis Molecular, Centro de Investigación y de Estudios Avanzados del Instituto Politécnico Nacional, Ciudad de Mexico 07360, Mexicomaritzaomanam@yahoo.com.mx (M.O.-M.); 5 Facultad de Estudios Superiores Iztacala, Medicina, Universidad Nacional Autónoma de México (UNAM), Tlalnepantla 54090, Mexico; 6 Instituto Universitario de Bio-Orgánica Antonio González (IUBO AG), Universidad de La Laguna (ULL), Avenida Astrofísico Francisco Sánchez 2, 38206 La Laguna, Spain; fcen@uv.mx (F.C.-P.); jjfercas@ull.edu.es (J.J.F.); 7 Facultad de Bioanálisis, Campus-Veracruz, Universidad Veracruzana, Veracruz 91700, Mexico; 8 Instituto de Productos Naturales y Agrobiología (IPNA), Consejo Superior de Investigaciones Científicas (CSIC), Avenida Astrofísico Francisco Sánchez 3, 38206 La Laguna, Spain; 9 Departamento de Química Orgánica, Universidad de La Laguna (ULL), Avenida Astrofísico Francisco Sánchez 3, 38206 La Laguna, Spain

**Keywords:** dehydrothyrsiferol, marine oxasqualenoid, *Leishmania amazonensis*, cutaneous leishmaniasis

## Abstract

One of the most important steps in preclinical drug discovery is to demonstrate the in vivo efficacy of potential leishmanicidal compounds and good characteristics at the level of parasite killing prior to initiating human clinical trials. This paper describes the use of dehydrothyrsiferol (DT), isolated from the red alga *Laurencia viridis*, in a pharmaceutical form supported on Sepigel, and the in vivo efficacy against a mouse model of cutaneous leishmaniasis. Studying the ultrastructural effect of DT was also carried out to verify the suspected damage at the cellular level and determine the severity of damages produced in the homeostasis of promastigotes. BALB/c mice infected with *Leishmania amazonensis* were divided into four groups: untreated mice, mice treated with miltefosine orally and mice treated topically with 1% and 0.5% DT-Sepigel; treatment was carried out for two weeks. Treatment with DT significantly reduced the parasite load in skin, liver and spleen compared with the untreated group. In addition, DT-Sepigel at the lowest concentration (0.5%) showed the best results, reducing lesion size by 87% at 3 weeks post-treatment. DT-Sepigel has demonstrated to be a potent topical treatment that, in combined drug trials, may aim at combating cutaneous leishmaniasis.

## 1. Introduction

Leishmaniasis is an infectious disease caused by the protozoan parasite of genus *Leishmania* and transmitted between animals and humans primarily through the bite of female insects of the Phlebotominae subfamily. Leishmaniasis includes a multitude of clinical manifestations that pose serious public health problems in endemic regions encompassing 98 countries. Among the three main forms of these infections, cutaneous leishmaniasis (CL) is the most common form, visceral leishmaniasis (VL) is the most severe form and mucocutaneous leishmaniasis (MCL) is the most disabling form of the disease [1].

Drug availability problems in endemic areas are exacerbated by the fact that most of these treatment regimens require parenteral administration, resulting in poor compliance, increased treatment costs and, ultimately, less favorable treatment outcome for patients. Moreover, a delayed diagnosis significantly worsens the efficacy of leishmaniasis treatments, exacerbating its outcome. The conventional gold standard treatment for the cutaneous form is mainly intralesional or parenteral administration of antimonials. Topical treatment is often combined with parenteral administration to achieve greater therapeutic efficacy. Several drugs, both new and old, have been reformulated for topical dosage forms and are under study to increase the therapeutic options for cutaneous leishmaniasis [2]. Miltefosine, the first oral drug for the treatment of this parasitosis, has also become an important alternative; however, its use is not yet licensed in many countries. While there is a limited selection of approved drugs for the treatment of leishmaniasis, most of them indeed active and effective in most cases, issues such as toxicity, cost and resistance pose challenges for some of these drugs [3]. However, several alternatives that include the use of natural products have been developed to treat cutaneous leishmaniasis, offering potential alternatives [4,5,6].

In terms of pharmaceutical formulations for topical application, gels are frequently used due to their capacity for controlled drug release. The primary challenge in gel development lies in maintaining the drug in a solubilized state while ensuring its controlled release. Recent research focused on gel-based formulations aims to improve drug penetration, retention and efficacy while minimizing systemic side effects [4]. Sepigel 305^TM^, a liquid polymer utilized as a gelling agent in cosmetics, is normally pre-neutralized and effective across a broad pH spectrum, serving as an exceptional stabilizer and texturizing agent. Displaying robust thickening capabilities in aqueous mediums, it stabilizes and emulsifies fatty phases across the board. Sepigel is composed of (i) the fatty oil isoparaffin, (ii) the gelation-promoting polymer polyacrylamide and (iii) the non-ionic emulsifier laureth-7 [5]. Due to polyacrylamide, the resulting gels promote wound healing by creating a moist environment, prevent wound desiccation and protect the wound from external contaminants by keeping the barrier moist and allowing oxygen and nutrients to reach the wound area, along with providing a Cushing’s effect that can minimize pain [6].

On the other hand, the study of the ultrastructural changes in the protozoa can provide insights into the mechanisms of action of therapeutic agents and the adaptive responses of these organisms, for instance, the alterations induced by certain treatments in cytoskeletal proteins and other proteins that play crucial roles in the life cycle of the protozoa [7]. These changes can have an impact on the organism’s ability to survive and reproduce, thereby influencing the course of infection and the effectiveness of the treatment. Research on the *Leishmania* genus, specifically *L. amazonensis*, has shown that certain compounds can lead to significant ultrastructural changes. Some treatments have been found to cause morphological alterations in the cell surface, a shortening of the flagellum, loss of mitochondrial matrix, disorganization of the kDNA and abnormal chromatin condensation or an increased number of autophagosomes, along with remarkable endoplasmic reticulum profiles surrounding different organelles [8], suggesting that autophagy, a physiological process of self-digestion of nonfunctional organelles and/or macromolecules, may play a crucial role in the response of *Leishmania* to drug treatment.

A large chemical arsenal of natural molecules with interesting undiscovered biological activities is hidden in the deep sea. This includes polyether compounds, such as the squalene-derived metabolites isolated from the red alga *Laurencia.* In fact, many triterpene polyether compounds with significant structural and pharmacological diversity have been identified. Within this family, compounds with different cytotoxic or inhibitory biological activities in the moderate to potent range have been described, including Ser-Thr type 2A protein phosphatase (PP2A) and integrin inhibitors [9,10,11,12,13].

**Figure 1:**
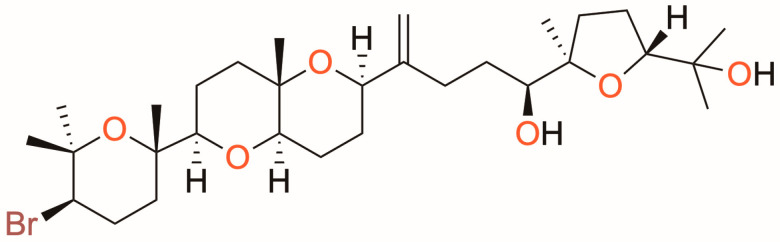
Chemical structure of dehydrothyrsiferol (DT).

The present study focuses on the evaluation of the oxasqualenoid dehydrothyrsiferol (DT, Figure 1), which was isolated from the red alga *Laurencia viridis* [14], and first reported in *L. pinnatifida* in 1984 [15]. In previous studies, DT was selected as a candidate molecule to analyze its mode of action, based on its proven trypanocidal and leishmanicidal activity (IC_50_ 9.45 ± 0.46 and 8.36 ± 0.77 μM against *T. cruzi* epimastigotes and *L. amazonensis* promastigotes, respectively). The activity of DT against the amastigote stage of *L. amazonensis* was even better than that of miltefosine, and it was confirmed that it induces apoptotic programmed cell death in this parasite strain [16]. Herein, DT was selected to explore the ultrastructural alterations produced on treated parasites, providing a comprehensive understanding of its effects. In addition, DT was tested in a gel formulation against an in vivo mouse model to assess the effects of topical administration as a potential treatment for cutaneous leishmaniasis.

## 2. Results

### 2.1. Ultrastructural Susceptibility of Promastigotes of Leishmania amazonensis to DT

**Figure 2:**
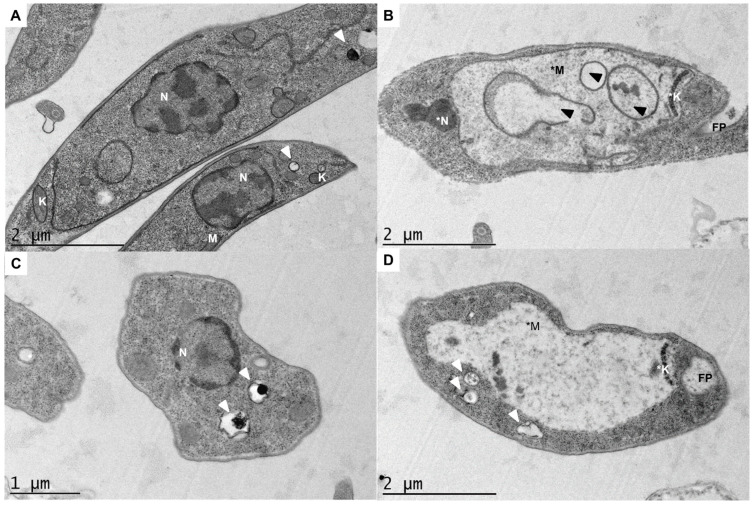
Transmission electron microscopy of L. amazonensis promastigotes after 24 h of incubation at the IC_90_ of DT. Parasite without treatment (image (**A**) showed its characteristic elongated body and normal morphology of the nucleus (N), mitochondrion (M), kinetoplast (K), flagellar pocket (FP) and acidocalcisomes (white arrows). DT-treated parasites (image (**B**–**D**) caused mitochondrial expansion and swelling (*M) and the formation of some concentric membrane structures inside the mitochondria (black arrows). We also can observe chromatin condensation in the nucleus (*N) and in the kinetoplast (*K). Moreover, larger acidocalcisomes with a higher density of their contents (white arrows) can be observed on the treated parasites, confirming the antikinetoplastid activity of the compound.

The transmission electron microscopy analysis of *L. amazonensis* promastigotes treated with DT was performed to determine the ultrastructural changes. Photomicrographs of promastigotes showed the degree of damage after 24 h of treatment compared with parasites without treatment, which showed normal morphology (Figure 2). The observation of *L. amazonensis* promastigotes treated with IC_90_ of DT showed clear damage to the parasite, with the appearance of a swollen organelle with concentric membrane structures inside it. The images showed that DT caused a great imbalance in the cellular homeostasis of the parasites, which hindered the development of their vital functions after 24 h of treatment, and could force the cell to a fatal outcome. It is notable that the mitochondrion was not visible, but we could see the slightly condensed DNAk (kinetoplast) inside the swollen organelle. Some authors have pointed out that important damages in the mitochondria, such as the fall of the electron transport chain, cause the same alterations in the mitochondrion of *Leishmania*, as, for example, in the case of Fe depletion already reported by Mesquita-Rodrigues and col., producing the same mitochondrial swelling and loss of cristae and matrices of the organelle [17]. This mitochondrial failure can also be corroborated by checking the drop in ATP production and mitochondrial potential in studies previously reported for DT-treated *L. amazonensis* [16].

Acidocalcisomes are specialized acidic organelles enriched with calcium and phosphorus found in Leishmania parasites. Although their function remains enigmatic, they are known to play an important role in the maintenance of intracellular pH homeostasis, due to their ability to uptake protons and regulate calcium and sodium ions, which contributes to the maintenance of the intracellular pH, making them crucial for the survival and virulence of the parasite [18,19]. In Figure 2B, some chromatin condensation inside the nucleus is detected, and, moreover, larger acidocalcisomes with a higher density of their contents can be observed after treatment of the parasites in Figure 2C,D.

### 2.2. In Vivo Evaluation of Topical DT-Sepigel Formulations in BALB/c Mice Infected by L. amazonensis

**Figure 3:**
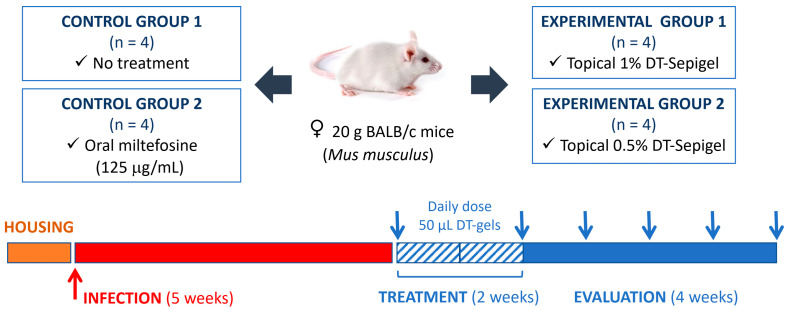
Scheme of treatment procedures used for the in vivo experiments. Blue arrows indicate the timeline of lesion measurement.

To evaluate the in vivo effects of DT topical treatment on cutaneous leishmaniasis, BALB/c mice were infected with *L. amazonensis* in the dermis at the base of the tail and treated daily with two different DT gels (1% and 0.5% Sepigel formulation) for two weeks, starting five weeks after infection (Figure 3). For this purpose, lesion size was monitored weekly.

**Figure 4:**
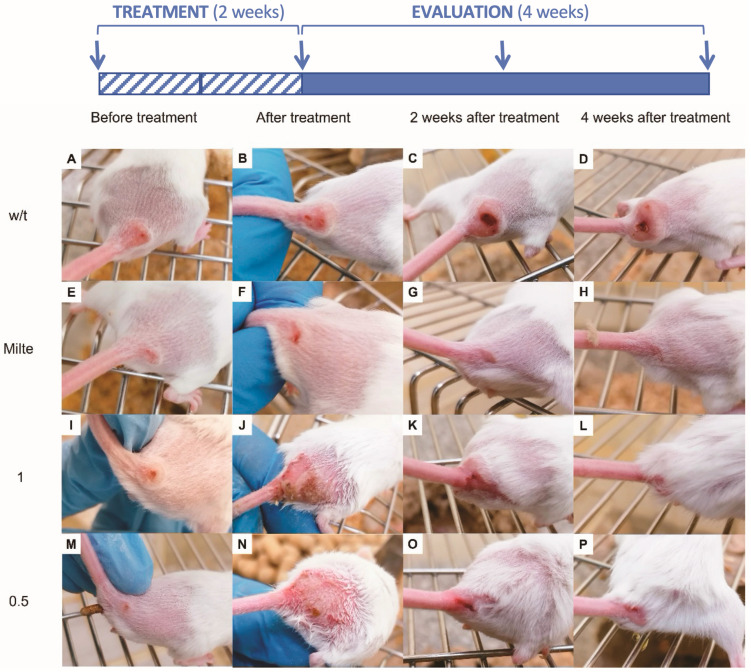
Efficacy of DT-Sepigel on cutaneous leishmaniasis (CL) lesions. Each row corresponds to a different treatment group: (**A**–**D**) Control 1: without treatment (w/t); (**E**–**H**) Control 2: oral miltefosine (Milte); (**I**–**L**) Experimental 1: topical 1% DT-Sepigel (1); and (**M**–**P**) Experimental 2: topical 0.5% DT-Sepigel (0.5). Each column corresponds to lesion measurement during the experiment (blue arrows): (**A**,**E**,**I**,**M**) dimension of the CL lesions before treatment; (**F**,**J**,**N**) dimension of the CL lesions after treatment (week 2); (**G**,**K**,**O**) dimension of the CL lesions 2 weeks after treatment (week 4), and (**H**,**L**,**P**) dimension of the CL lesions 4 weeks after treatment (week 6).

The analysis of lesion size (Figure 4) showed that the growth rate of lesion size decelerated during the treatment with DT and showed a difference between the groups receiving 1% or 0.5% of DT, with the group treated at the lowest concentration responding much better to treatment. DT treatment significantly reduced lesion size compared with untreated mice 5 weeks after the start of treatment.

In the untreated control group, the lesions grew in size, the inflammation increased and even ulcerations appeared, while, in the experimental groups treated with DT, neither great inflammation nor too much growth of the lesion were observed. Moreover, the small ulcerations that emerged in the lesions were reduced over the weeks until complete disappearance at week 4 post-treatment. In the case of the mice control group treated with oral miltefosine, the most striking and different effect observed was the lack of inflammation of the lesions, although small ulcerations appeared, which also disappeared after 4 weeks.

### 2.3. Evaluation of DT Treatment in the Evolution of Skin Lesions in Mice Infected with L. amazonensis

**Figure 5:**
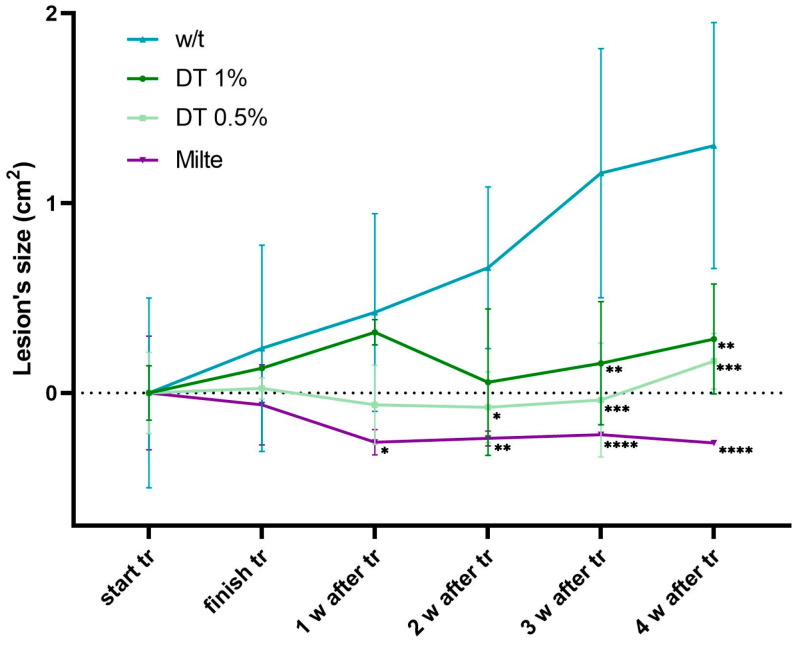
Progression of lesion size in *L. amazonensis*-infected BALB/c mice treated with different concentrations of topical DT-Sepigel. Lesion size was measured in two dimensions using calipers, and the mean lesion diameters were determined. To normalize the data, the mean lesion sizes were taken as zero for each group, in order to see the evolution per group, with respect to their baseline. w/t—without treatment; Milte—oral miltefosine; DT 1%—topical 1% DT-Sepigel; DT 0.5%—topical 0.5% DT-Sepigel; tr—treatment; w—week. * *p* < 0.05, ** *p* < 0.01, *** *p* < 0.001 and **** *p* < 0.0001.

The evolution of lesion size in animals infected with *L. amazonensis* was monitored from the 1st day of treatment until 4 weeks after finishing the treatment. Lesion size represents the average area of the lesion in cm^2^ (n = 4). The data of mean lesion sizes at the beginning of the treatment were taken to be zero, to be able to better compare lesion evolutions between groups (Figure 5).

DT-treated animals showed a decrease in lesion size relative to the untreated group, which was most significant at 4 weeks after the end of treatment. Two weeks after the end of treatment, the DT-treated groups began to show a decrease in lesion diameter area relative to the untreated group of mice. The miltefosine-treated group showed an even greater difference compared with the untreated control in lesion measurements. However, if only the group of mice that received the 0.5% DT treatment is considered, it was evident that the trend was quite similar to that of the mice treated with oral miltefosine, except in the fourth week after treatment, where the lesion size seemed to increase, probably because of a possible recurrence due to the parasites that remained alive in the lesions. Comparison between 1% and 0.5% DT-Sepigel treatments revealed that, although the concentration at 0.5% seemed to decrease lesion sizes more efficiently in the first week after treatment, the lesion sizes between the two doses of DT seemed to approach each other from the second to the fourth post-treatment week.

Figure 5 shows that the reductions in lesion sizes are significant compared with the untreated control at 2 weeks post-treatment (*p* = 0.0232 for 0.5% DT), at 3 weeks post-treatment (*p* = 0. 0014 for 1% DT and *p* = 0.0002 for 0.5% DT) and 4 weeks post-treatment (*p* = 0.0012 for 1% DT and *p* = 0.0003 for 0.5% DT).

### 2.4. Evaluation of Parasite Burden in Treated Mice by Quantitative PCR

**Figure 6:**
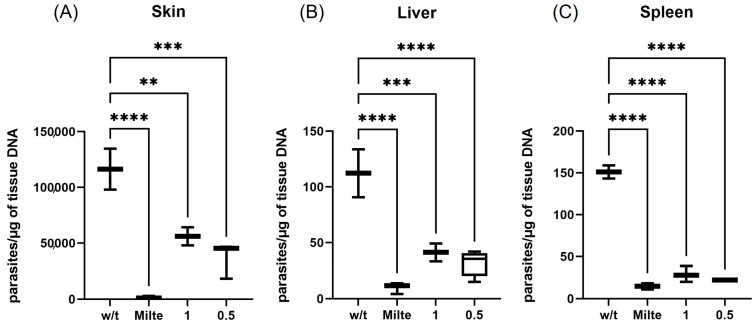
Skin, liver and splenic parasite burden in BALB/c mice treated with topical DT-Sepigel. The number of *L. amazonensis* parasites was quantified using qPCR from tissue of (**A**) skin (lesion edge), (**B**) liver and (**C**) spleen tissue from the control and experimental groups of mice at week 4 after the end of treatment. w/t—without treatment; Milte—oral miltefosine; 1—topical 1% DT-Sepigel; 0.5—topical 0.5% DT-Sepigel. ** *p* < 0.01, *** *p* < 0.001 and **** *p* < 0.0001.

To investigate the efficacy of DT-Sepigel as a topical treatment against *L. amazonensis*, the parasite load of the skin (lesion edge), spleen and liver was also assessed and compared with the oral treatment with miltefosine (Figure 6). Animals treated with both 1% and 0.5% DT-Sepigel showed a significant reduction in parasite burden compared with the untreated control group. Of note, a slight inverse concentration–effect relationship was observed in the case of the three studied tissues: skin, liver and spleen. The lower concentration of DT (0.5%) showed a greater effect in reducing the parasite load in these tissues compared with the higher concentration of DT (1%), thus demonstrating that a lower dose produces a similar or even greater leishmanicidal effects.

Figure 6 shows that the reduction in parasite load in the skin (lesion) was significant compared with the untreated control (*p* = 0.0011 DT 1%, and *p* = 0.0002 DT 0.5%), while the parasite loads in liver and spleen showed a greater significance (*p* < 0.0003 for DT 1% and *p* < 0.0001 DT 0.5% in liver) (*p* < 0.0001 for DT 1% and DT 0.5% in spleen) compared with the effect produced by miltefosine (*p* < 0.0001 in skin, liver and spleen).

## 3. Discussion

DT, an oxasqualenoid compound isolated from the red alga *Laurencia viridis*, has been selected as a lead molecule due to its antikinetoplastid effects against *L. amazonensis*, *L. donovani* and *T. cruzi*. It is noteworthy that the activity of DT against the amastigote stage of *L. amazonensis* was even better than miltefosine. In addition, DT was found to produce a type of programmed cell death with chromatin condensation and damage at the mitochondrial level, such as a decrease in mitochondrial potential and subsequent reduction in ATP levels [16]. Furthermore, in previous work, the pharmacokinetic properties of DT and its ADME/Tox were predicted in silico, resulting in good solubility, permeability, drugability and metabolism parameters [20].

In this study, electron microscopy experiments have shown that DT causes clear damage to the parasites, and a slight condensation of the kinetoplast and nucleus. Furthermore, the appearance of a swollen organelle with membrane structures are suggestive that it may be the mitochondrion, corroborating previous results regarding the mitochondrial damage produced by DT in promastigotes of *L. amazonensis*. These intracellular homeostasis problems are also reflected in the increase in size of the acidocalcisomes, organelles in charge of the regulation of some homeostasis processes and pH regulation.

Based on previous results [16,20], the topical use of DT-Sepigel is proposed in this work as a possible alternative for the treatment of CL, and compared with oral treatment with the reference drug, miltefosine. Concentrations of 1% and 0.5% DT-Sepigel demonstrated to be effective in mice infection of *L. amazonensis*. Among the two experimental groups, the group treated with DT-Sepigel at the lowest concentration (0.5%) showed the best results, reducing lesion size by 87% at 3 weeks post-treatment. Furthermore, treatment with DT significantly reduced the parasite load in skin, liver and spleen compared with the untreated group. Interestingly, this study revealed a remarkable disparity in parasite load between miltefosine and DT-Sepigel treatment groups, particularly in skin, showing comparable results to miltefosine in liver and spleen. The systemic nature of the treatment with miltefosine may have played a key role in the decrease in the parasite load observed. However, DT-Sepigel was also able to reduce this load, thus demonstrating its ability to prevent visceralization of the infection. Therefore, the results highlight the potential of this topical treatment, not only in terms of reducing toxicity but also for its ability to effectively prevent the visceralization of the infection.

Similar studies evaluating the in vivo activity of natural compounds fail to eliminate the infection completely. Although some studies do manage to reduce the parasitic load better than the reference treatment, very few studies use miltefosine as the reference drug instead of other older and more toxic drugs such as pentavalent antimonials or amphotericin B [21]. For instance, Bilbao-Ramos et al. managed to reduce lesion sizes by 42% after 10 weeks post-treatment with ursolic acid [22], while Araújo et al. reduced the number of parasites in the lesion approximately 24.5 times lower than in the untreated group [22]. In another study, (*E*)-piplartin achieved a reduction of around 35% in lesions with respect to the untreated group after 50 days post-treatment [23]. In the present study, a reduction of lesions of 82% after two weeks post-treatment and 87% after three weeks post-treatment was obtained using the lowest tested concentration of DT-Sepigel (0.5%).

This therapeutic approach highlights two important advantages: one primarily concerning treatment adherence and the other pertaining to toxicity profiles. On the one hand, most treatments for cutaneous leishmaniasis involve intravenous, intramuscular or even intralesional administration. Therefore, the use of a less invasive treatment will always favor treatment adherence, hence its completion and consequent improvement in the control of leishmaniasis from an epidemiological standpoint [24], and almost all the existing treatments for leishmaniasis are systemic treatments, which are known to show remarkable systemic toxicity, such as teratogenicity, nephrotoxicity, hepatotoxicity and gastrointestinal problems [25]. The proposed topical treatment would prevent all these toxicities, avoiding its excessive passage through the bloodstream, thus highlighting its potential for safer clinical application.

## 4. Materials and Methods

### 4.1. Isolation and Purification of Dehydrothyrsiferol (DT)

Specimens of *Laurencia viridis* were collected off the coast of Paraiso Floral (Tenerife, Canary Islands; 28°07′12″ N, 16°46′45″ W) and extracted with CHCl_3_:MeOH (1:1) at room temperature. After solvent evaporation, a dark-green viscous crude extract was chromatographed on a Sephadex LH-20 (Merck Life Science S.L., Madrid, Spain) column (7 × 50 cm) using CH_3_Cl/MeOH (1:1). The fraction enriched in DT was fractionated on a silica gel column (7 × 50 cm) using a linear gradient of *n*-hexane/EtOAc (4:1―1:4) to obtain the major component. The compound was purified by recrystallization in a mixture of *n*-hexane/CH_2_Cl_2_. The spectroscopic data of the pure compound confirmed the structure of DT (see Supporting Information) [15].

### 4.2. Parasites

*Leishmania amazonensis* promastigotes (MHOM/BR/77/LTB0016) were cultivated in Schneider (Sigma-Aldrich, St. Louis, MO, USA) medium supplemented with 10% fetal bovine serum (FBS; Gibco, Madrid, Spain and 10 μg/mL of gentamicin (Sigma, Barcelona, Spain) at 26 °C. Daily counts of parasites culture was performed in an image-based cytometer Tali (Invitrogen by Life Technologies, Madrid, Spain). In vitro passages of culture were performed upon reaching the stationary phase of growth and the infectivity of the parasites was maintained through passages in BALB/c mice.

### 4.3. Animals

Female 6- to 12-week-old BALB/c mice (*Mus musculus*) were raised and maintained at the animal facilities of the Universidad de La Laguna, San Cristóbal de La Laguna, Tenerife, Spain. Animals were maintained in rooms with controlled temperature (22 ± 2 °C), humidity (55 ± 10%), continuous air renovation, 12:12 light/dark cycle, balanced diet for rodents and water *ad libitum*. The protocol was approved by the Research Ethics and Animal Welfare Committee of Universidad de La Laguna (authorization approval number: CEIBA2019-0367) according to European Guidelines [26].

### 4.4. Ultrastructural Analysis

The ultra structures of *L. amazonensis* treated with DT and control promastigotes were analyzed by transmission electron microscopy (TEM). First, promastigotes of *L. amazonensis* were incubated with the IC_90_ of DT (29.06 µM) for 24 h at 26 °C. After incubation, the cells were fixed for 1 h at room temperature with a solution of 2.5% glutaraldehyde in sodium cacodylate buffer 0.1 M, pH 7.2 (Electron Microscopy Sciences, Hatfield, PA, USA). Subsequently, the cells were washed with sodium cacodylate buffer 0.1 M, pH 7.2 four times, and post-fixation was performed with a solution of 1% osmium tetroxide (Sigma Chemical Co., St. Louis, MO, USA) in the same buffer for 1 h. Cells were dehydrated in increasing concentrations of ethanol (50, 70, 90 and 100%) during 10 min for each step, and finally embedded in epoxy resin Poly/Bed 812 (PolyScience Inc., Warrington, PA, USA). Thin sections (60 nm) were obtained, collected, contrasted with uranyl acetate and lead citrate and analyzed in a JEOL JEM-1011 transmission electron microscope (JEOL Ltd., Tokyo, Japan) [27].

### 4.5. DT-Sepigel Formulation

For in vivo treatment, a gel-based formulation containing 1% (35 mg of DT in 3500 µL) and 0.5% (17.5 mg of DT in 3500 µL) (*w*/*v*) of DT in Sepigel 305™ (Acofarma, Madrid, Spain) was prepared. First, the active compound was weighed and dissolved in DMSO to a maximum volume of 1% for complete distribution. Next, Sepigel 305™ was added under continuous stirring to obtain a homogeneous gel formulation. The prepared gels were stored at 4 °C until use [28].

### 4.6. In Vivo Infection and Treatment Administration

BALB/c mice (female, 5–7 weeks old, around 20 g) were infected at the base of the tail with 1 × 10^6^ *L. amazonensis* promastigotes at a stationary phase in 50 μL of physiological saline solution. After 5 weeks of infection, mice were treated daily for 2 weeks with 50 µL of the DT gels by topical route, containing 1% and 0.5% of the active compound, covering the whole lesion/ulcer/inflammation. Drug administration for mice treated orally with miltefosine was carried out by dissolving the drug in their drinking water, taking into account the average water intake dose according to the weight of the mice [29]. The development of lesions was monitored weekly using a caliper (Figure 2). The size of the lesion was determined by the calculation of the area, sizing the thickness and length of the lesion. Mice were sacrificed one month after the end of treatment, and skin (lesion), liver and spleen were collected for parasite quantification.

### 4.7. DNA Extraction

Skin, liver and spleen tissues of the infected mice, treated and untreated with DT gels, were subjected to a DNA extraction procedure. After the mice were sacrificed, two skin biopsies of the lesion were taken with a standard biopsy punch, and the liver and spleen were removed and frozen. The biopsy specimens were cut and weighed to be subsequently subjected to TissueRuptor (Quiagen, Singapore), followed by lysis with proteinase K and DNA isolation using the Illustra TM Tissue & Cells genomicPrep Mini Spin kit (GE Healthcare, Singapore).

### 4.8. Parasite Quantification

Parasite quantification was estimated by qPCR. For each reaction, 2 μL DNA extract samples (0.5 ng/μL) were amplified with 5 μL of Power SYBR Green PCR Master Mix (AppliedBiosystems, Waltham, MA, USA) and 5 μM of each primer in a total volume of 10 μL. For the qPCR reaction, 40 cycles were carried out, where the steps of each cycle stage were 20 s at 95 °C, 20 s at 60 °C and 20 s at 72 °C. The final step of a melting curve was set up as 15 s at 95 °C, 1 min at 60 °C and 15 s at 95 °C. This was set up in a Step One Plus (ThermoFisher Scientific, Waltham, MA, USA) real-time PCR machine. The pair of primers used were LMi-amaF—AAAATGAGTGCAGAAACCC and MLR—CGGCCCTATTTTACACCAACC′, which amplified a fragment of the kDNA (qPCR-ama) (adapted from Diotallevi et al. [30]). A standard curve with serially diluted known concentrations (10 ng, 1 ng, 0.1 ng, 0.01 ng, 0.001 ng) of parasite DNA equivalents/reaction was performed for *L. amazonensis* (MHOM/BR/77/LTB0016) (slope = 3.265; R^2^ = 0.98; E = 102.4%). All clinical tissues and standard samples were run in duplicate. A positive control sample of a known amount of *Leishmania* parasites was used.

To compare the data obtained from each tissue sample with the standard curve, the amount of *Leishmania* was calculated as follows: [parasite DNA equivalents per reaction/amount of tissue DNA per reaction] × 10^3^, expressed as the number of *Leishmania* parasites per mg of tissue DNA, considering the size of the *L. amazonensis* haploid genome 83.15 fg of DNA as one parasite equivalent.

### 4.9. Statistical Analysis

The data are displayed as mean ± standard deviation (SD) derived from four independent experiments, representing the outcomes consistently obtained. Statistical variance between means was assessed employing a one-way analysis of variance (ANOVA) for four samples, followed by Tukey’s test for pairwise comparisons of means, facilitated by SigmaPlot 12.0 software and GaphPad 9.0 software. A significant level of *p* < 0.05 was considered for all analyses.

## 5. Conclusions

The topical use of the marine oxasqualenoid, dehydrothyrsiferol (DT), supported by Sepigel, is proposed in this work as a possible alternative for the treatment of CL. In this in vivo study, concentrations of 1% and 0.5% DT-Sepigel demonstrated to be effective using a mouse model of CL. Among the two experimental groups, the group treated with DT-Sepigel at the lowest concentration (0.5%) showed the best results, reducing lesion size by 87% at 3 weeks post-treatment. Furthermore, treatment with DT significantly reduced the parasite load in skin, liver and spleen compared with the untreated group. Interestingly, this study revealed a remarkable disparity in parasite load between miltefosine and DT-Sepigel treatment groups, particularly in skin, showing comparable results to miltefosine in liver and spleen.

In order to deepen the mode of action of DT, electron microscopy experiments showed clear damage to promastigotes of *L. amazonensis*, and a slight condensation of the kinetoplast and nucleus. Furthermore, the appearance of a swollen organelle with membrane structures was suggestive that it may be the mitochondrion, corroborating previous results regarding the mitochondrial damage. These intracellular homeostasis problems were also reflected in the increase in size of the acidocalcisomes, organelles in charge of the regulation of some homeostasis processes and pH regulation.

All of these findings suggest an important step forward in the development of DT-Sepigel as a topical treatment option in combination with drug studies targeting cutaneous leishmaniasis for more effective therapeutic strategies.

## Data Availability

The original contributions presented in the study are included in the article, further inquiries can be directed to the corresponding author.

## Supplementary Materials

The following supporting information can be downloaded at: https://www.mdpi.com/article/10.3390/md23010013/s1, Table S1. ^1^H and ^13^C NMR data of dehydrothyrsiferol in CDCl_3_ at 298 K, 600 MHz; Figure S1. ^1^H-NMR spectrum of dehydrothyrsiferol in CDCl_3_ at 298 K, 600 MHz; Figure S2. ^13^C NMR spectrum of dehydrothyrsiferol in CDCl_3_ at 298 K, 150 MHz; Figure S3. HSQC spectrum of dehydrothyrsiferol in CDCl_3_ at 298 K, 600 MHz; Figure S4. MS spectrum of dehydrothyrsiferol.
