# Supplementary material for: Dehydrothyrsiferol Against Cutaneous Leishmaniasis: Treatment Outcome in a Murine Model
Source: Mar Drugs. 2024 Dec 28;23(1):13. doi: 10.3390/md23010013 (PMC11766930; doi:10.3390/md23010013)

Supporting information

Dehydrothyrsiferol Against Cutaneous Leishmaniasis: Treatment Outcome in a Murine Model

Atteneri López-Arencibia[1,2,3], Carlos J. Bethencourt-Estrella[1], Desirée San Nicolás-Hernández[1], Rubén L. Rodríguez-Expósito[1], Angélica Dominguez-Barrios[1], Lizbeth Salazar-Villatoro[4], Maritza Omaña-Molina[4,5], Francisco Cen-Pacheco[6,7], Ana R. Díaz-Marrero[6,8], José J. Fernández[6,9], A. Elizabeth Córdoba-Lanús[1,2], Jacob Lorenzo-Morales[1,2,3], José E. Piñero[1,2,3]

1 Instituto Universitario de Enfermedades Tropicales y Salud Pública de Canarias (IUETSPC), Universidad de La Laguna (ULL), Avenida Astrofísico Francisco Sánchez s/n, 38206 La Laguna, Tenerife, Spain
2 Consorcio Centro de Investigación Biomédica en Red M.P. de Enfermedades Infecciosas (CIBERINFEC), Instituto de Salud Carlos III, 28006 Madrid, Spain
3 Departamento de Obstetricia y Ginecología, Pediatría, Medicina Preventiva y Salud Pública, Toxicología, Medicina Legal y Forense y Parasitología, Universidad de La Laguna (ULL), Tenerife, Spain
4 Departamento de Infectómica y Patogénesis Molecular, Centro de Investigación y de Estudios Avanzados del Instituto Politécnico Nacional, Ciudad de Mexico 07360, Mexico
5 Facultad de Estudios Superiores Iztacala, Medicina, Universidad Nacional Autónoma de México (UNAM), Tlalnepantla 54090, Mexico
6 Instituto Universitario de Bio-Orgánica Antonio González (IUBO AG), Universidad de La Laguna (ULL), Avenida Astrofísico Francisco Sánchez 2, 38206 La Laguna, Tenerife, Spain
7 Facultad de Bioanálisis, Campus-Veracruz, Universidad Veracruzana, Veracruz 91700, Mexico
8 Instituto de Productos Naturales y Agrobiología (IPNA), Consejo Superior de Investigaciones Científicas (CSIC), Avenida Astrofísico Francisco Sánchez 3, 38206 La Laguna, Tenerife, Spain
9 Departamento de Química Orgánica, Universidad de La Laguna (ULL), Avenida Astrofísico Francisco Sánchez 3, 38206 La Laguna, Tenerife, Spain

Table of contents

**Table S1**	^{1}H and ^{13}C NMR data of dehydrothyrsiferol in $CDCl_3$ at 298 K, 600 MHz.	**S2**
**Figure S1**	^{1}H-NMR spectrum of dehydrothyrsiferol in $CDCl_3$ at 298 K, 600 MHz.	**S3**
**Figure S2**	^{13}C NMR spectrum of dehydrothyrsiferol in $CDCl_3$ at 298 K, 150 MHz.	**S4**
**Figure S3**	HSQC spectrum of dehydrothyrsiferol in $CDCl_3$ at 298 K, 600 MHz.	**S5**
**Figure S4**	MS spectrum of dehydrothyrsiferol.	**S6**

Table S1. ^1H and ^{13}C NMR data of dehydrothyrsiferol in $CDCl_3$ at 298 K, 600 MHz.

Carbón	δ ^{13}C	δ ^1H
**1**	31.0	1.27
**2**	74.9	
**3**	59.0	3.89
**4**	28.2	(α)2.13/2.26(β)
**5**	37.1	(α)1.52/1.80(β)
**6**	74.4	
**7**	86.7	3.08
**8**	22.9	(β)1.49/1.76(α)
**9**	38.7	(α)1.44/1.77(β)
**10**	72.9	
**11**	78.9	3.43
**12**	21.8	(β)1.60/1.81(α)
**13**	26.6	(β)1.86/2.11(α)
**14**	72.5	4.28
**15**	151.3	
**16**	29.9	2.12/2.43
**17**	30.3	1.45/1.63
**18**	76.2	3.52
**19**	86.0	
**20**	31.7	(α)1.60/2.12(β)
**21**	26.3	1.84(2H)
**22**	87.6	3.76
**23**	70.5	
**24**	23.9	1.12
**25**	23.6	1.39
**26**	20.1	1.20
**27**	19.4	1.23
**28**	109.8	4.88/5.05
**29**	23.7	1.14
**30**	27.7	1.21

Figure S1. ^{1}H-NMR spectrum of dehydrothyrsiferol in $CDCl_3$ at 298 K, 600 MHz.

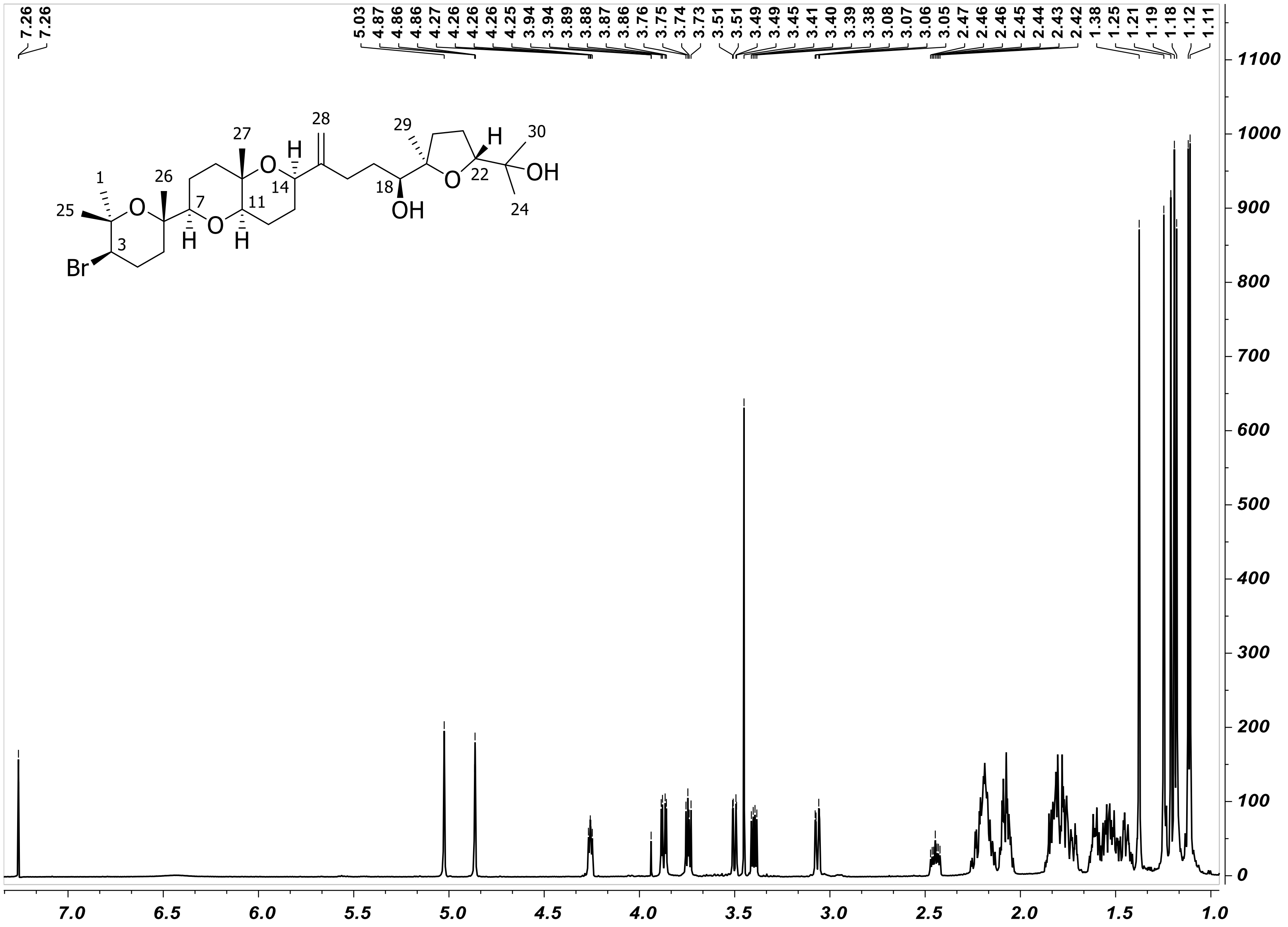

Figure S2. ^{13}C NMR spectrum of dehydrothyrsiferol in $CDCl_3$ at 298 K, 150 MHz.

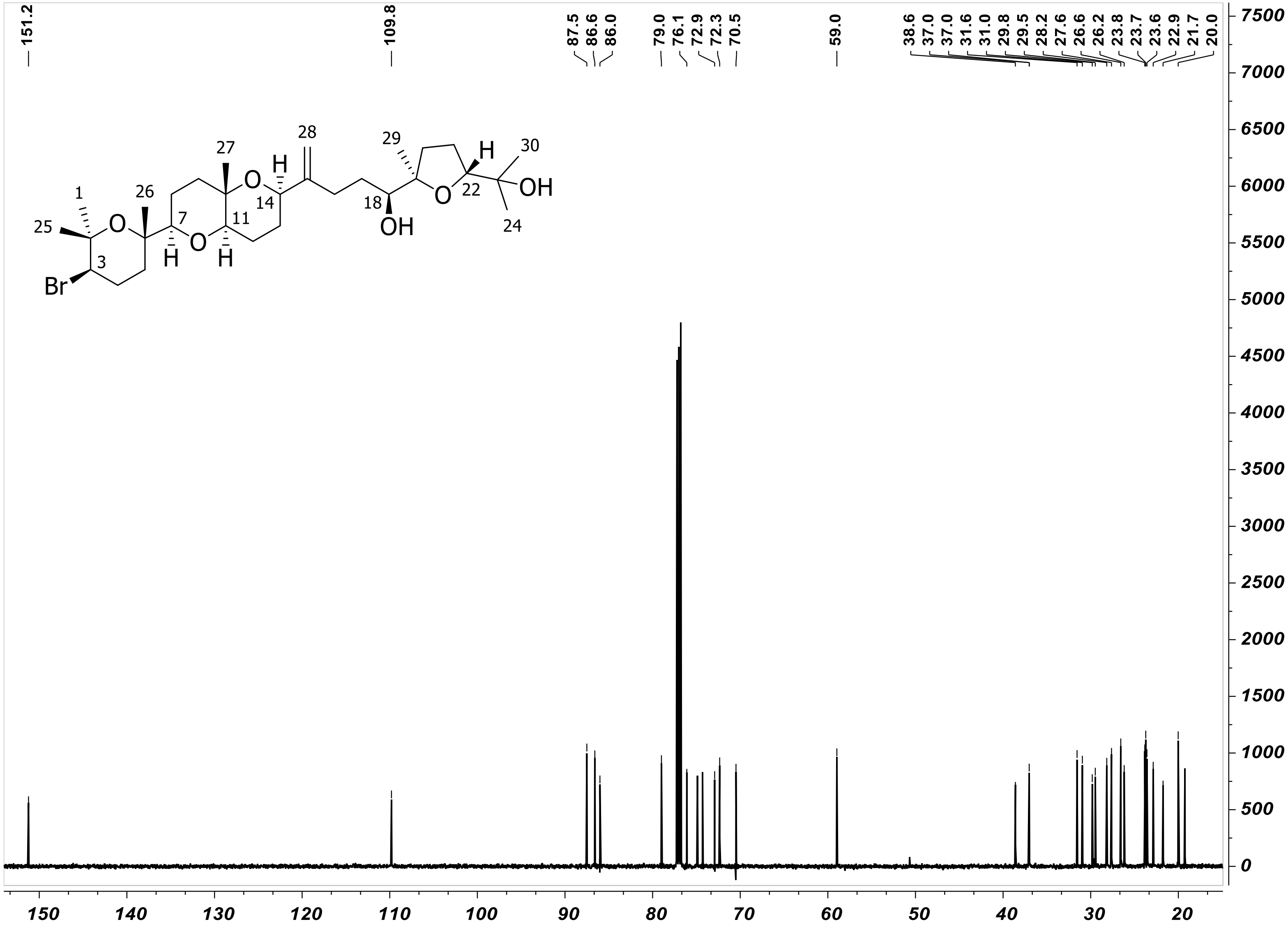

Figure S3. HSQC spectrum of dehydrothyrsiferol in $CDCl_3$ at 298 K, 600 MHz.

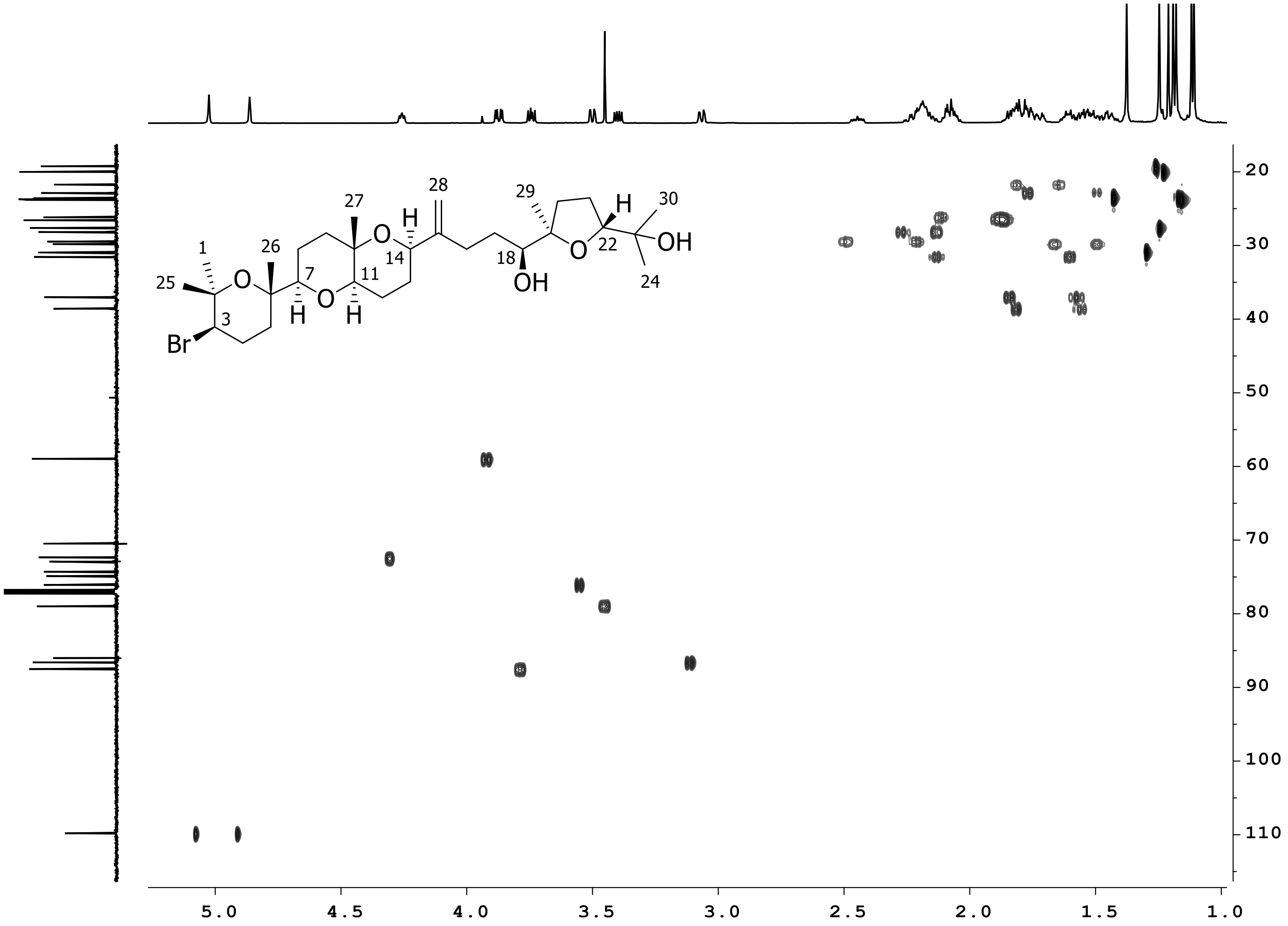

Figure S4. Mass spectrum of dehydrothyrsiferol.

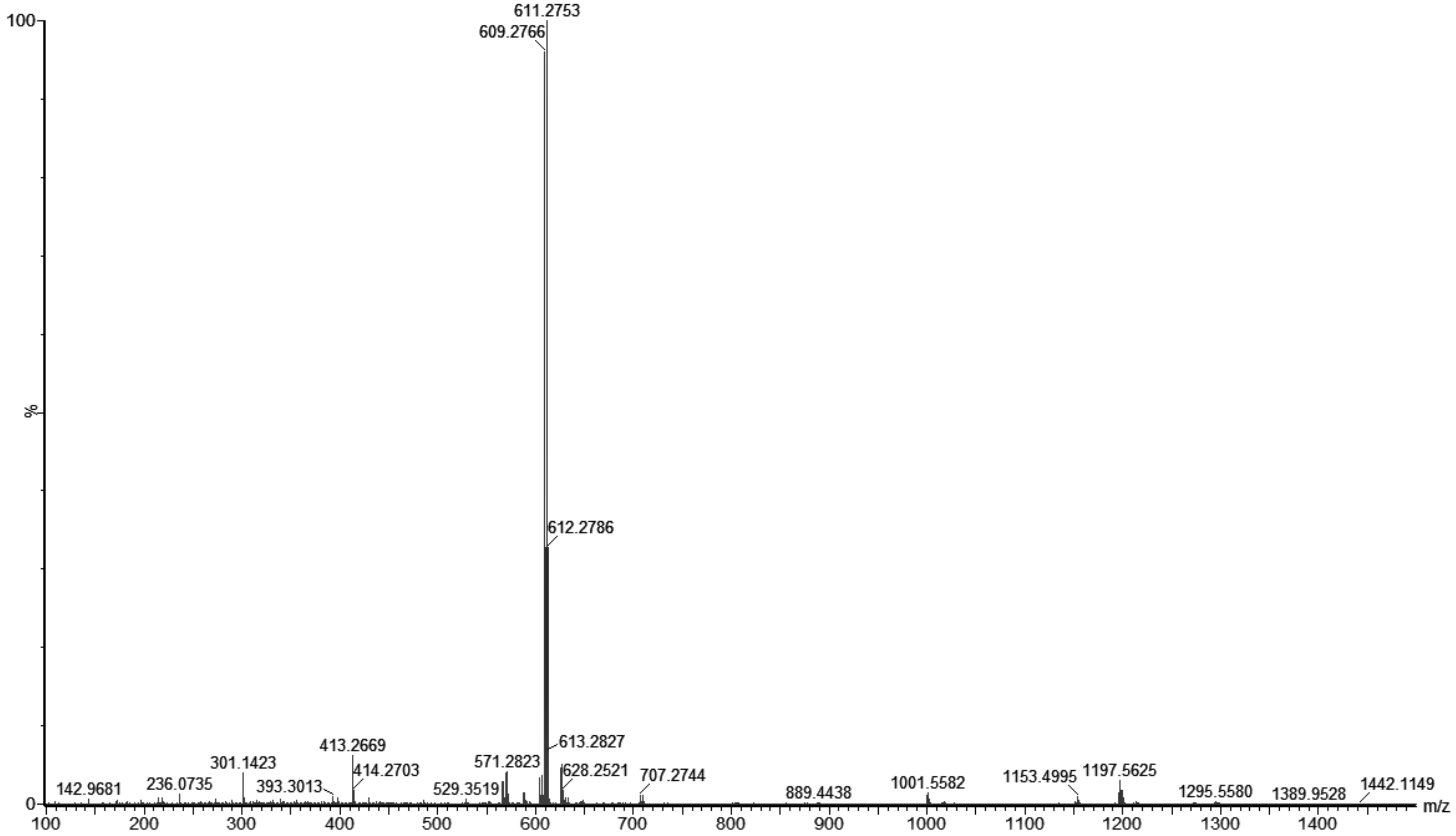

Supplement: Supplementary file 1 [file marinedrugs-23-00013-s001.zip › marinedrugs-3343551-supplementary.pdf]
